# A Simple and Rapid Methicillin-Resistant *Staphylococcus aureus* (MRSA) Screening Test Using a Mannose-Binding Lectin (MBL)-Conjugated Gold Nanoparticle Probe

**DOI:** 10.4014/jmb.2301.01004

**Published:** 2023-03-09

**Authors:** So Yeon Yi, Jinyoung Jeong, Wang Sik Lee, Jungsun Kwon, Kyungah Yoon, Kyoungsook Park

**Affiliations:** 1 Bionanotechnology Research Center, Korea Research Institute of Bioscience and Biotechnology (KRIBB), Daejeon 34141, Republic of Korea; 2 Environmental Disease Research Center, Korea Research Institute of Bioscience and Biotechnology (KRIBB), Daejeon 34141, Republic of Korea; 3 Department of Bioengineering, KRIBB School, University of Science and Technology, Daejeon 34141, Republic of Korea; 4 BioNano Health Guard Research Center, Daejeon 34141, Republic of Korea; 5 Department of Clinical Pathology, Daejeon Health Institute of Technology, Daejeon 34504, Republic of Korea; 6 Department of Biopharmacy, Daejeon Health Institute of Technology, Daejeon 34504, Republic of Korea

**Keywords:** Antibacterial susceptibility testing, gold nanoparticles, mannose-binding lectin, methicillin-resistant *Staphylococcus aureus*, screening

## Abstract

Rapid diagnosis of methicillin-resistant *Staphylococcus aureus* (MRSA) is essential for guiding clinical treatment and preventing the spread of MRSA infections. Herein, we present a simple and rapid MRSA screening test based on the aggregation effect of mannose-binding lectin (MBL)-conjugated gold nanoparticles (AuNP), called the MRSA probe. Recombinant MBL protein is a member of the lectin family and part of the innate immune system. It can recognize wall teichoic acid (WTA) on the membrane of MRSA more specifically than that of methicillin-sensitive *Staphylococcus aureus* (MSSA) under optimized salt conditions. Thus, the MRSA probe can selectively bind to MRSA, and the aggregation of the probes on the surface of the target bacteria can be detected and analyzed by the naked eye within 5 min. To demonstrate the suitability of the method for real-world application, we tested 40 clinical *S. aureus* isolates (including 20 MRSA specimens) and recorded a sensitivity of 100%. In conclusion, the MRSA probe-based screening test with its excellent sensitivity has the potential for successful application in the microbiology laboratory.

## Introduction

*Staphylococcus aureus* (*S. aureus*) is one of the most common opportunistic human pathogens. It can cause a variety of diseases ranging from mild skin infections to serious and life-threatening infections, such as pneumonia, meningitis, endocarditis, osteomyelitis, and sepsis [1]. Recently, the emergence of multidrug-resistant *S. aureus*, in particular, methicillin-resistant *S. aureus* (MRSA), has become a severe threat to public health, and its related health costs have increased dramatically. In addition, MRSA is frequently resistant to various antibiotics and therefore poses a problem in the treatment of infectious diseases [2, 3].

The conventional MRSA detection methods in the clinic are culture-based antimicrobial susceptibility testing (including disc diffusion, broth dilution assays, and the Epsilometer test (E-Test), which are all labor-intensive and time-consuming. Molecular diagnostic techniques have been developed as alternative approaches, and these include qPCR, antibody-based ELISA, and nucleic acid hybridization [4]. More recently, rapid diagnostic techniques such as immuno-PCR, mass spectrometric analysis, and biosensor techniques have also been reported [5, 6]. However, these methods still have limitations due to their requiring trained personnel and expensive instruments. Thus, a rapid, simple, and accurate method to detect MRSA is urgently needed.

Mannose-binding lectin (MBL) is a critical host-defense protein that activates the lectin complement pathway of the innate immune system [7, 8]. As a calcium-dependent, broad-spectrum opsonin, MBL can bind to microbial surface carbohydrate molecules, referred to as pathogen-associated molecular patterns (PAMPs) [9], including lipopolysaccharide endotoxin (LPS), wall teichoic acid (WTA), and lipoteichoic acid (LTA) [10-13]. MBL reportedly binds to PAMPs on the surfaces of many different types of pathogens, including gram-negative and gram-positive bacteria, and pathogen detection methods using MBL, such as enzyme-linked immunosorbent assays (ELISA), immunofluorescence, and scanning electron microscopy (SEM) have also been developed [14-18].

However, numerous discrepancies are reportedly associated with MBL binding, depending on the method used, and various laboratories have also reported different binding affinities [19-21]. As described previously, MBL can bind to carbohydrate moieties (mannose and *N*-acetyl-glucosamine) embedded in the surface of microorganisms. The *S. aureus* cell wall is a complex structure composed of capsular polysaccharides, WTA, LTA, peptidoglycan, and lipoproteins. Generally, MBL is known to recognize and bind to both α- and β-GlcNAc residues of WTA present in the *S. aureus* cell wall in a Ca^2+^-dependent manner [22-24]. However, MBL has a higher affinity for β-GlcNAc WTA [23]. Furthermore, a recent study reported that WTA has important roles in the resistance to antimicrobial molecules, host interactions, virulence, and biofilm formation, and that β-glycosylation of WTA is critical for the resistance of MRSA to β-lactam [25, 26]. Mistretta, N *et al*. demonstrated the stress-induced environmental influences of WTAs, including that of being induced in high salt culture conditions, leading to β-glycosylation [26].

**Scheme 1:**
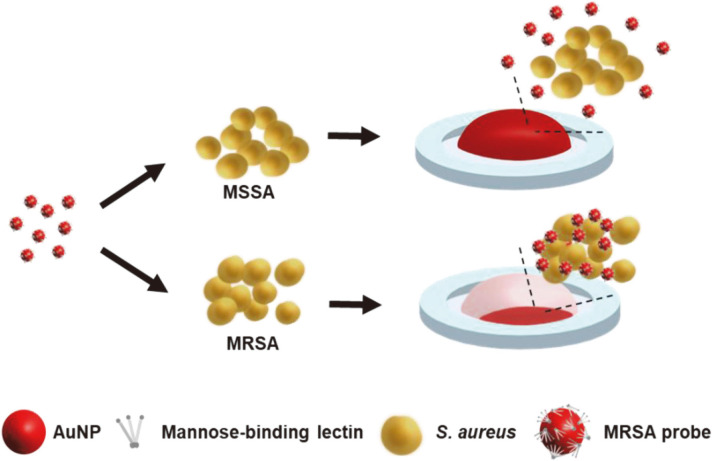
Schematic illustration of the MRSA screening test using MBL-AuNP conjugates, called the MRSA probe.

In this study, we developed an MRSA screening test using MBL-conjugated gold nanoparticles (AuNP), called the MRSA probe, to detect MRSA in a simple and rapid manner. The experimental procedure is schematically described in Scheme 1. The MRSA probe was created by conjugating MBL with AuNPs, which involved simply mixing them together. When the MRSA probe was mixed with MRSA, MBL was able to bind to the surface of the MRSA, and the aggregation of AuNPs could be observed with the naked eye. To increase the specific binding ability to MRSA, *S. aureus* was grown in a low salt condition. Visually, 5 min after sample addition, the MRSA screening test was able to accurately detect whether a specimen was MRSA or not. To validate the MRSA test, 40 clinically isolated *S. aureus* samples were utilized, and the results showed a sensitivity of 100%. Therefore, we expect that this simple and quick MRSA screening test will enable naked-eye detection of MRSA within 5 min, thereby providing rapid clinical decision in the management of MRSA.

## Materials and Methods

### Bacterial Cultures and Growth Conditions

The *Escherichia coli* (*E. coli*, ATCC 25922), *Pseudomonas aeruginosa* (*P. aeruginosa*, ATCC 27853), *Klebsiella pneumonia* (*K. pneumonia*, ATCC 13883), *Shigella flexneri* (*S. flexneri*, ATCC 29903), and *Staphylococcus aureus* (*S. aureus*, ATCC 6538P, MSSA) used in this research were obtained from the Korean Collection for Type Cultures (KCTC, Korea). *Enterococcus faecium* (*E. faecium*, ATCC 19434), *Staphylococcus epidermidis* (*S. epidermidis*, ATCC 14990), and *S. aureus* (ATCC 29213) were purchased from the Korean Culture Center of Microorganisms (KCCM, Korea). *S. aureus* (ATCC 29213), which is MSSA, was the type strain. In this study, we used *S. aureus* (ATCC 6538P) as a negative control for the tests since it was MSSA. *Salmonella enterica* (*S. enterica*, ATCC 15277), MRSA (ATCC 43300), and *E. coli* O157:H7 (ATCC 35150) were provided by the American Type Culture Collection (ATCC, USA). In a total of 40 clinical *S. aureus* isolates (including 20 MRSA and 20 MSSA), 20 samples (MRSA 77~86 and MSSA 85~94) were kindly provided by Prof. D. Yong of the Yonsei University College of Medicine (Korea) and 20 samples (MRSA 11485, 11486, 11487, 11488, 11489, 14754, 15867, 15868, 15871, 15872, MSSA 11495, 11857, 11862, 14747, 14753, 14755, 15917, 15918, 15919, 15920) were obtained from the National Culture Collection for Pathogens (NCCP, Korea). The twenty clinical samples (MRSA 77~86 and MSSA 85~94) from Prof. D. Yong were tested using the broth dilution method to determine the antimicrobial susceptibility and obtain MIC values in our previous study [27].

All colony-purified strains were minimally passaged and stored at −80°C in Luria-Bertani broth (LB, BD Diagnostics, USA) in 15% glycerol (Sigma-Aldrich, USA) before use in this study. All of the other chemicals were of analytical grade, and purified water was produced by a Millipore water purification system (EMD Millipore, USA).

### Gene Cloning, Overexpression, and Purification of the Recombinant MBL Protein

Synthetic mannose-binding lectin DNA (GenBank #BC096179) with a c-terminal six histidine tag (6× His-tag) was obtained from Bioneer (Korea). The MBL DNA was cloned into pAcGP67A (Pharmingen, USA) to create the pAcGP67A–MBL-6× His–tag. To express the protein, *Trichoplusia ni* BTI–TN–5B1–4 (High Five) insect cells (Invitrogen, USA) were grown in Insect Hi-Express serum-free media before infection with the high titer virus. High Five insect cells were used to express the MBL after infection with the recombinant baculovirus.

To purify the recombinant MBL, 1 L of recombinant baculovirus-infected insect cell culture was harvested approximately 72 h post-infection and the cells were pelleted by centrifugation at 5,000 ×*g* for 15 min. The supernatant was dialyzed against binding buffer (50 mM Tris-HCl, pH 8.0, 0.5 M NaCl) and loaded onto a Ni^2+^-nitrilotriacetic acid (Ni-NTA) agarose column (Qiagen, Germany) equilibrated with the same buffer. The supernatant was then washed with binding buffer containing 50 mM imidazole. The MBL was eluted with 500 mM imidazole in binding buffer and its presence was monitored by sodium dodecyl sulfate-polyacrylamide gel electrophoresis (SDS-PAGE) throughout the purification. The purified MBL migrated as approximately 26 kDa proteins on SDS-PAGE, which corresponded to their molecular weight. The eluted glycoproteins were dialyzed with Slide-A-Lyzer (3.5 K MWCO; Thermo Scientiﬁc, USA) in phosphate-buffered saline (PBS, pH 7.4) overnight at 4°C, and stored in PBS (pH 7.4) or 5% (v/v) glycerol at –70°C. The protein concentrations were determined via the Bradford method, using bovine serum albumin as a standard.

### Preparation of the MRSA Probe

The MRSA probe, which is the recombinant MBL protein-conjugated gold nanoparticle, was prepared as follows: AuNPs were synthesized by reducing gold (III) chloride using trisodium citrate, and 60 mg of HAuCl_4_ dissolved in 125 ml of water was boiled. Then, 25 ml of 1% trisodium citrate was added to the HAuCl_4_ solution with stirring, and boiled for 20 min, during which the solution became red.

After cooling, 40 μg of protein was added to 1 ml of AuNP solution (0.1 mg/ml), which was incubated for 2 h at 25°C. One microliter of 2% BSA was then added and the solution was further incubated at 4°C overnight to stabilize the conjugate. The unbound proteins and BSA were removed by centrifugation (15,000 ×*g*, 20 min) three times with 0.1× PBS (pH 7.4). Finally, the conjugate was dissolved in reaction buffer (20 mM Tris HCl pH 7.4, 100 mM NaCl, 10 mM CaCl_2_, 10 mM MgCl_2_) for use.

### Characterization of the MRSA Probe

The morphological properties of the AuNP and the MRSA probe were analyzed using a 200 kV field-emission transmission electron microscope (FE-TEM, JEM-2100F, JEOL Ltd., Japan). A droplet of 10 μg/ml AuNP and the MRSA probe dispersions were dropped on the Formvar/Carbon grid (TED PELLA, Inc., USA) and dried in a fume hood. The size distributions of the AuNP and MRSA probes were analyzed from the images by using ImageJ.

The hydrodynamic size and zeta-potential values were characterized by a particle size analyzer (Zetasizer Nano ZS, Malvern Instruments Ltd., UK). Disposable folded capillary cells (DTS1070, Malvern Instruments Ltd.) were used for the hydrodynamic size and zeta-potential determination. AuNP and MBL-AuNP (the MRSA probe) were prepared in desterilized water and Tris buffer (pH 7.4), respectively.

### Bacterial Binding Affinity Test of the MRSA Probe

For the strain selectivity assay [28, 29], culture broth (LB) was inoculated from colonies of each of the indicated bacterial species. The bacteria were grown in a shaking incubator for 16~24 h and then washed in PBS. We then mixed 50 ml of the MRSA probe with 50 ml of bacteria (1 × 10^6^ cells/ml) and incubated them for 5 min.

For optimization of the MRSA screen test, the bacteria were grown overnight (~16 h) at 37°C on a solid agar plate supplemented with 0.5% yeast extract, 1% tryptone, and a NaCl concentration of 0.5, 1, or 2% v/v. Two or three colonies of the bacteria were individually placed on the plastic cards and then one drop (50 ml) of the MRSA probe was added. The bacteria and the MRSA probe were mixed using a rod. We found visible aggregates on the card within 5 min by naked-eye detection when MRSA was used.

### Scanning Electron Microscopic Image

To visualize the cell surface binding of the MRSA probe on the surface of MRSA, FE-SEM was applied. MRSA or MSSA were simply mixed with the MRSA probes at RT for 5 min. After the washing steps, the samples were fixed in 4% v/v formaldehyde solution in PBS for 1 h, dehydrated with DW, and mounted on a Si-wafer. The samples were observed using a field-emission scanning electron microscope (FE-SEM, Quanta 250 FEG, FEI, USA) with an electron beam of 15 kV on low damage mode.

### MRSA Screening Test with Clinical Samples

After 16 h incubation on agar plates supplemented with 0.5% yeast extract, 1% tryptone, and 0.5% NaCl, bacterial colonies were picked and streaked on the plastic cards. Then, the MRSA probe (1 drop; 50 ml) was placed on the plastic cards and mixed with the bacterial cells. A total of 40 clinical *S. aureus* isolates, including 20 MRSA and 20 MSSA, were tested. MRSA (ATCC 43300) was used as a positive control in this study. *S. aureus* (ATCC 29213) and *S. aureus* (ATCC 6538P; MSSA) were used as negative controls for the MRSA screening test. After 5 min for the reaction, the aggregation was observed visually and compared with the positive and negative controls.

### Data Analysis

Sensitivity was defined as the percentage of positive results where the sample was truly positive, and specificity was defined as the percentage of negative results where the sample was truly negative. All formulas were as follows, and TP, TN, and FP represent the true positive, true negative, and false positive, respectively:

Sensitivity = TP/(TP+TN)

Specificity = TN/(TN+FP)

## Results and Discussion

### Preparation and Characterization of the MRSA Probe

**Fig. 1:**
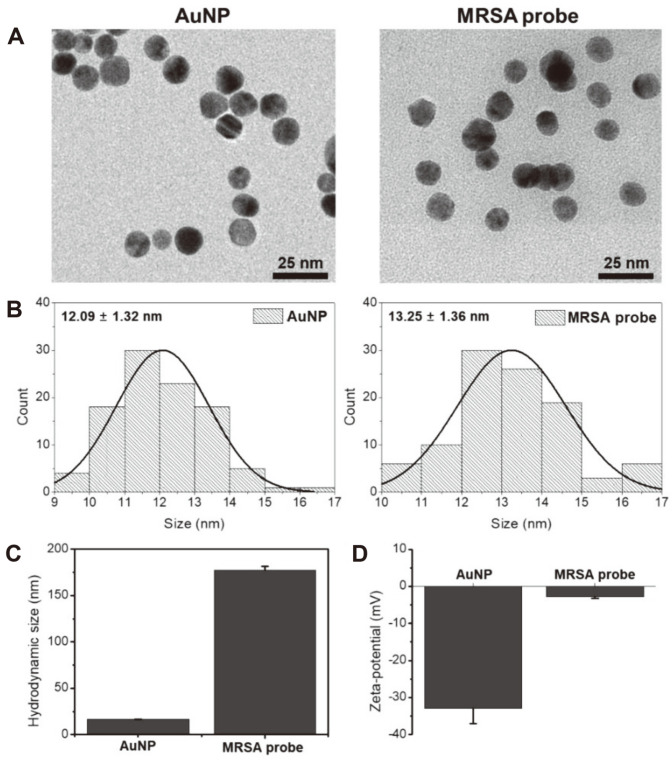
Characterization of MRSA probe. The MRSA probe was characterized by FE-TEM, DLS and zeta-potential. (**A**) TEM images and (**B**) size distribution (*n* = 100) of the AuNP and the MRSA probe. (**C**) The hydrodynamic size and (**D**) zeta-potential values of the AuNP and the MRSA probe measured by DLS.

To prepare the MRSA probe, we first over-expressed and purified recombinant MBL protein using an insect cell expression system. The AuNPs were simply prepared by citrate reduction, which is a common method, and showed strong absorbance with red color for application in naked-eye detection (Fig. S1). The purified recombinant MBL proteins were conjugated with the AuNPs by simply mixing and incubating them for 2 h at 25°C without the need for any other chemical reactions. After the washing steps, we could clearly see the conjugated recombinant MBL on AuNP by SDS-PAGE gel with MBL concentrations at 10, 20, 30, and 40 mg/ml, respectively (Fig. S2A). Moreover, the MBL-AuNPs with different MBL concentrations were pretested to determine the aggregation by mixing with MRSA. As a result, the aggregates were formed distinctively as the MBL concentration increased compared to the non-aggregated AuNP without MRSA (Fig. S2B). Thus, 40 mg/ml MBL-conjugated AuNP was selected as the concentration for the MRSA probe in this study. We first characterized the physical properties of the MRSA probe by the dynamic size scattering (DLS) and FE-TEM. Figs. 1A and 1B showed the TEM images and size distribution of the AuNPs and the MRSA probe, both of which were homogenously spherical in shape with an average size of 12.09 ± 1.32 nm and 13.25 ± 1.36 nm, respectively. However, the hydrodynamic size and zeta-potential were clearly different before and after MBL conjugation. The hydrodynamic size of the AuNP was 16.62 ± 0.05 nm and the polydispersity index (PDI) was 0.138 ± 0.02. For the MRSA probe, the hydrodynamic size and PDI increased to 177.47 ± 4.19 nm and 0.346 ± 0.01, respectively (Fig. 1C). Additionally, the zeta-potential value of the AuNPs increased from −32.93 ± 4.05 to −2.75 ± 0.44 after the recombinant MBL proteins were conjugated on the surface of the AuNPs (Fig. 1D). We assumed that the conjugation of recombinant MBL proteins on AuNP led to the nanoparticles’ becoming neutral charged from negatively charged, resulting in the increase of the hydrodynamic size of the MRSA probe due to low ionic strength.

### Binding Affinity Tests of Recombinant MBL Proteins Using Dot Blot Assays

To confirm the binding efficiency of MBL against various bacterial strains, we conducted dot blot assays. As previously described, MBL binding to various bacteria has been demonstrated. However, a mismatch of MBL binding can appear according to the experimental methods. Furthermore, the efficiency of MBL binding to living bacteria depends on the differences in encapsulation within the same bacterial genus and species [25].

For the dot blot assay, various bacteria were spotted onto nitrocellulose (NC) membranes and incubated with the MBL proteins. After washing, the MBL binding was detected by an enhanced chemiluminescence (ECL) assay. As shown in Fig. S3, the MBL showed different binding affinity against various bacteria. For MRSA, the binding signal was stronger than against other types of bacteria. Weak signals were detected against *S. aureus* and *P. aeruginosa*, and no signal was measured against the other bacteria, including *E. coli*, *S. flexneri*, *K. pneumonia*, *B. cereus*, *S. enterica*, *S. haemolyticus*, *S. saprophytic*, *E. faecalis*, *E. faecium*, and *S. pneumonia*. These results demonstrate that the MBL binding affinity is dependent on the bacterial strain. Of note, we found that MBL was able to bind to MRSA much more strongly than to any of the other species. To determine the limit of detection (LOD), we mixed MBL proteins with various concentrations of *S. aureus*. The LOD was less than 1 × 10^6^ cells/ml (Fig. S3B).

### Optimization of the *S. aureus* Growth Conditions for the MRSA Screening Test

**Fig. 2:**
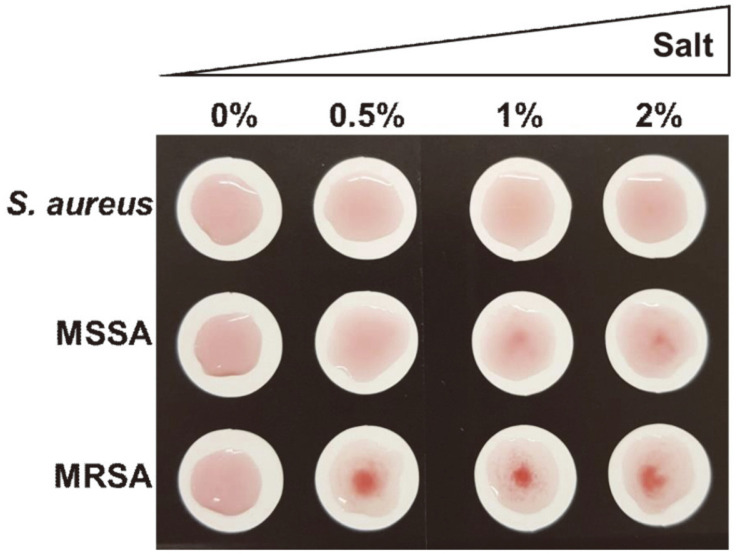
The salt effect on the MRSA screen test. Image showing different aggregations of the MRSA probe vs. the salt concentration in the culture medium.

Based on Mistretta’s report, a high salt culture condition provided a stressed environment for the bacteria, and WTA led to β-glycosylation [26]. In high salt conditions, β-glycosylation is increased in both MSSA and MRSA. To enhance the selectivity of the MRSA screening test, we optimized the *S. aureus* growth conditions, such as the salt concentration. After *S. aureus* was grown under different salt concentrations (0.5, 1, or 2%) on agar plates, the MRSA probe was mixed and the aggregations were observed. Fig. 2 shows that the MRSA-specific aggregation was well detected, even among those growing in the low salt growth condition of a 0.5% NaCl agar plate, while no aggregations were detected with MSSA that grew under the same condition. Thus, we believe that growth in low salt media further clarifies the distinctions of the MRSA screening test since it influences cell wall changes in both MSSA and MRSA.

### Characterization of the MRSA Probe Selective Binding Using SEM

**Fig. 3:**
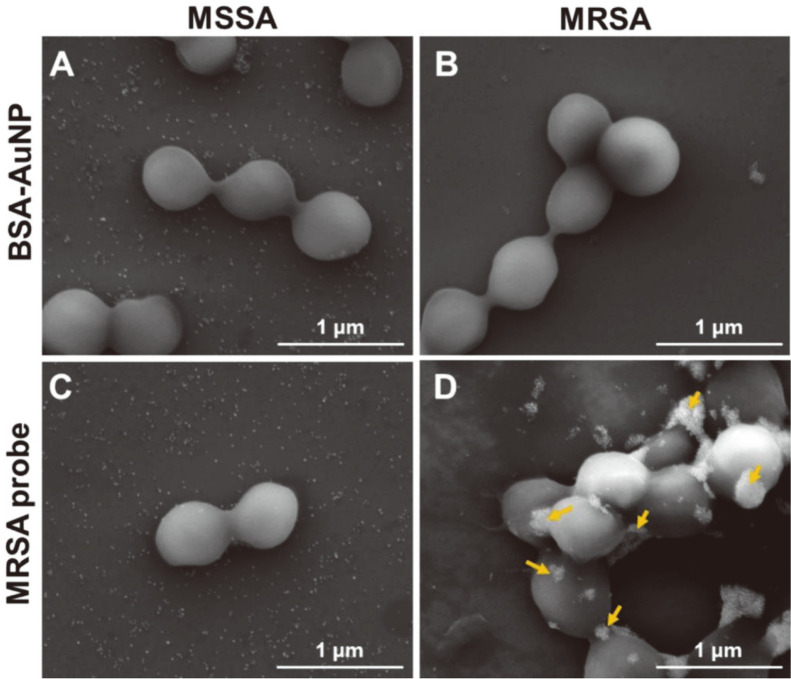
Selective binding test using FE-SEM analysis. SEM images after performing the BSA-AuNP aggregation assay with (**A**) MSSA and (**B**) MRSA. SEM images after performing the MRSA probe aggregation assay with (**C**) MSSA and (**D**) MRSA. Yellow arrows indicate the MRSA probes.

Fig. 3 shows the SEM images of MSSA and MRSA after mixing with BSA-AuNP and the MRSA probe for 5 min. BSA-AuNP was used as negative control which conjugated BSA, instead of MBL. As seen in Figs. 3A and 3B, BSA-AuNPs were not seen on the surface of either MSSA or MRSA. Similarly, the MRSA probe did not attach to the surface of MSSA in Fig. 3C, and only the aggregated form was distinctively observed on the MRSA probe mixed with MRSA in Fig. 3D. These results demonstrated that the aggregation of the MRSA probe occurred only in the presence of MRSA. This assay does not require any preprocessing steps for the bacterial samples and they can be directly adsorbed by the MRSA probe within 5 min.

### Binding Test of the MRSA Probe Against Various Bacteria

**Fig. 4:**
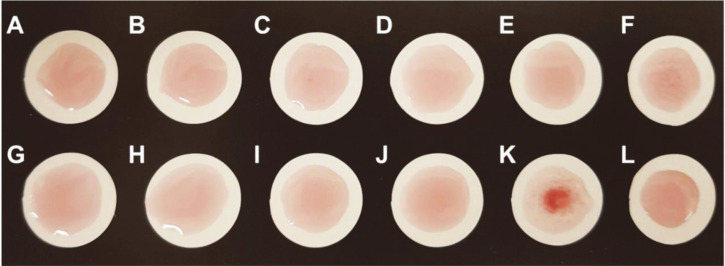
Selectivity test. Images were obtained after the aggregation assay using MRSA probes with the bacterial samples containing (**A**) *E. coli*, (**B**) *E. coli* O157:H7, (**C**) *P. aeruginosa*, (**D**) *K. pneumonia*, (**E**) *S. flexneri*, (**F**) *S. enterica*, (**G**) *E. faecium*, (**H**) *S. epidermidis*, (**I**) *S. aureus* (ATCC 29213), (**J**) *S. aureus* (ATCC 6538P, MSSA), (**K**) *S. aureus* (ATCC 43300, MRSA) typical strongly positive reaction, and (**L**) negative control within 5 min. Negative control means without a bacterial sample in this test.

To perform the aggregation of the MRSA probe against various bacteria strains, we also tested the binding efficiency of the MRSA probe. For the test, the MRSA probe was simply mixed and incubated with three or more colonies of various bacteria on a plastic card (Fig. 4). Various types of bacteria, such as *E. coli*, *E. coli* O157:H7, *P. aeruginosa*, *K. pneumonia*, *S. flexneri*, *S. enterica*, *E. faecium*, *S. epidermidis*, *S. aureus* (ATCC 29213), *S. aureus* (ATCC 6538P, FMSSA), and MRSA were used. We identified the bacterial cell numbers to determine the sensitivity of the method for naked-eye detection (Fig. S4). In this study, we used *S. aureus* (ATCC 6538P) as a negative control for the tests representing MSSA. As seen in Fig. 4, only the samples containing MRSA showed the formation of red precipitates on the plastic card 5 min after adding the MRSA probe. In the case of MSSA, the weak red precipitate formation was detected after 10 min (Fig. S5). However, the negative control and the other bacteria, including *P. aeruginosa*, did not show any precipitates on the plastic card after 10 min. These results demonstrated that the sedimentation rate of the MRSA probe-MRSA complex was accelerated and visualized faster than any other bacterial complex.

### Validation of the MRSA Screening Test with Clinical Samples

**Fig. 5:**
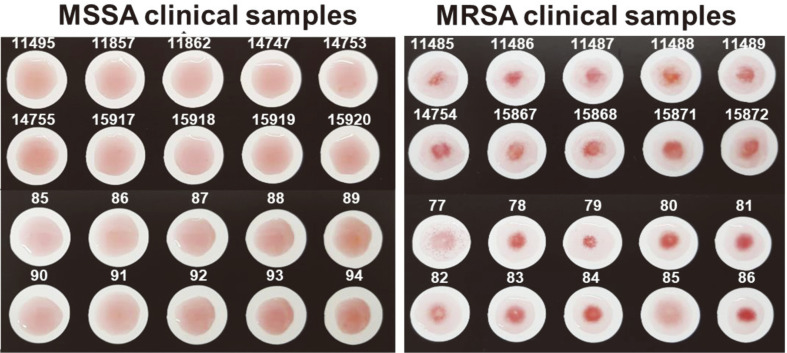
MRSA screening test with 40 clinical *S. aureus* isolates. The results showed MRSA specific aggregation when the MRSA probe was mixed with 20 MRSA isolates, while MSSA showed no aggregation, with the exception of two MSSA isolates (89 and 94), which showed weak responses against the MRSA probe.

To validate the MRSA screening test, 40 clinical *S. aureus* isolates, including 20 MRSA, were employed. After simply mixing the samples with the MRSA probe, the results were observed by naked-eye detection within 5 min. As shown in Fig. 5, 20 of the clinical MRSA isolates showed aggregation, and 18 of 20 MSSA did not. Two of the 20 MSSA showed weak aggregation in this test. The sensitivity and specificity of the MRSA screening test were revealed to be 100% and 95%, respectively (Table 1). This result suggested that the MRSA screening test is a quick and simple method, requiring no devices, and could be scored with the naked eye. Of course, considering the small number of strains that were tested, this difference may alternatively be explained by chance. These results require further validation of the assay’s potential applications in bacterial monitoring.

In this study, we have demonstrated a new-concept MRSA screening test using MBL-AuNPs conjugates, called the MRSA probe, for distinguishing between MSSA and MRSA by naked-eye detection. Visually, 5 min after sample addition, the MRSA screening test made it possible to detect whether the sample was MRSA or MSSA. To validate the MRSA screening test, we utilized 40 clinical isolates of *S. aureus*, including 20 MRSA, and the results showed a sensitivity of 100%. Therefore, we expect that this simple and rapid MRSA screening test will enable the identification of MRSA by naked-eye detection within 5 min, thus greatly reducing the time required for clinical treatment decisions.

## Supplemental Materials

Supplementary data for this paper are available on-line only at http://jmb.or.kr.

## Figures and Tables

**Table 1:** Sensitivity and specificity of the MRSA screening test against clinical *S. aureus* isolates.

MRSA (n = 20)		MSSA (n = 20)		Sensitivity (%)	Specificity (%)

TP	FN	TP	FP		
20	0	18	2	100 %	95 %
