# Supplementary material for: A Simple and Rapid Methicillin-Resistant Staphylococcus aureus (MRSA) Screening Test Using a Mannose-Binding Lectin (MBL)-Conjugated Gold Nanoparticle Probe
Source: J Microbiol Biotechnol. 2023 Mar 9;33(5):698–705. doi: 10.4014/jmb.2301.01004 (PMC10243892; doi:10.4014/jmb.2301.01004)
Supplement: Supplementary file 1 [file jmb-33-5-698-supple.pdf]

J. Microbiol. Biotechnol.
https://doi.org/10.4014/jmb.2301.01004

Supplementary Figures

A simple and rapid methicillin-resistant *Staphylococcus aureus* (MRSA) screening test using mannose-binding lectin (MBL)-conjugated gold nanoparticle probe

So Yeon Yi[1†], Jinyoung Jeong[2,3†], Wang Sik Lee[2], Jungsun Kwon[4], Kyungah Yoon[5], and Kyoungsook Park[6*]

[1] Bionanotechnology Research Center, Korea Research Institute of Bioscience and Biotechnology (KRIBB), 125 Gwahak-ro, Yuseong-gu, Daejeon 34141, Republic of Korea

[2] Environmental Disease Research Center, Korea Research Institute of Bioscience and Biotechnology (KRIBB), Daejeon 34141, Republic of Korea

[3] Department of Bioengineering, KRIBB School, University of Science and Technology, Daejeon 34141, Republic of Korea

[4] BioNano Health Guard Research Center, Daejeon 34141, Republic of Korea

[5] Department of Clinical Pathology, Daejeon Health Institute of Technology, Daejeon 34504, Republic of Korea

[6] Department of Biopharmacy, Daejeon Health Institute of Technology, 21 Chungjeong-ro, Dong-gu, Daejeon 34504, Republic of Korea

Materials and Methods

Bacterial Strains

E. coli (KCTC 1116), *Acinetobacter baumannii* (*A. baumannii*, ATCC 19606), *P. aeruginosa* (ATCC 10145), *Bacillus cereus* (*B. cereus*, ATCC 14579), *S. aureus* (ATCC 25923), *Staphylococcus haemolyticus* (*S. haemolyticus*, ATCC 29970), *Staphylococcus saprophyticus* (*S. saprophyticus*, ATCC 15305), *Enterococcus faecalis* (*E. faecalis*, ATCC 19433), *E. faecalis* (ATCC 29212), and *Vibrio vulnificus* (*V. vulnificus*, ATCC 27562) used in this research were distributed by the Korean Collection for Type Cultures (KCTC, Korea). *E. faecalis* (ATCC 51299), *Entercocuus faecium* (*E. faecium*, ATCC 700221), *Bordetella parapertussis* (*B. parapertussis*, ATCC 15311), and *K. pnneumoniae* (ATCC 700603) were provided from the American Type Culture Collection (ATCC, USA). *Streptococcus pneumonia* (*S. pneumonia*, ATCC 33400) was purchased from the Korean Culture Center of Microorganisms (KCCM, Korea). *V. vulnificus* was grown on Marine broth at, 30℃ and *B. parapertussis* was grown on Bordet Gengou medium. All colony-purified strains except *V. vulnificus* and *B. parapertussis* were minimally passaged and stored at −80°C in Luria-Bertani broth (LB, BD Diagnostics, USA) in 15 % glycerol (Sigma-Aldrich, USA) before use in this study.

Sodium Dodecyl Sulfate-Polyacrylamide Gel Electrophoresis (SDS-PAGE) and Western Blot Analysis

The MBL-AuNPs were separated on NuPAGE Novex 4–12 % Bis–Tris SDS-PAGE gels in MES-SDS running buffer (50 mM MES, 50 mM Tris–base, 0.1 % SDS, 1 mM EDTA, pH 7.3; Invitrogen) and visualized by staining with coomassie brilliant blue R-250. Gel images were obtained using a gel documentation system (AE–9000 E–graph, ATTO Corp.).

Evaluating the Interaction of Mannose-Binding Lectin (MBL) and Various Bacteria the Target by Overlay Assay

Two microliters of various bacteria (1×10^6 Cells/mL) were spotted onto the Hybond C-extra NC membrane (Bio-Rad, USA) next to each other. The membrane was blocked for 1 h at 25℃ in tris-buffered saline containing 0.05 % Tween-20, 10 mM $CaCl_2$, and 5 % skim milk. The membrane was then incubated with MBL (10 μM) at 4℃ overnight. The membrane was washed three times for 20 min at each wash. The MBL overlaid membrane was incubated with anti-6x His Tag antibody for 1 h at 25℃. After the five times washing, HRP-conjugated anti- rabbit IgG was treated for 1 h at 25℃. The interaction was detected by measuring the enhanced chemiluminescence (ECL kit, Thermo Fisher Scientific, USA).

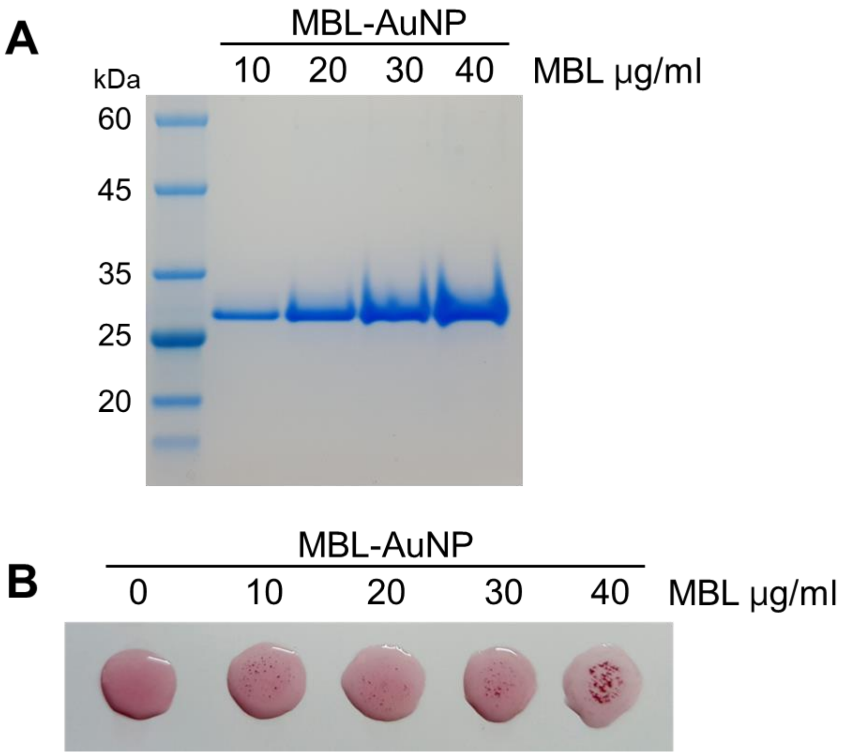

Figure S1. AuNP conjugation image according to MBL concentration. (A) Image showed the degree of conjugation to gold particles by MBL concentration using SDS-PAGE. (B) Image where the degree of aggregation was visually confirmed by mixing MRSA probe for each concentration with MRSA (1×10^6 cells/ml).

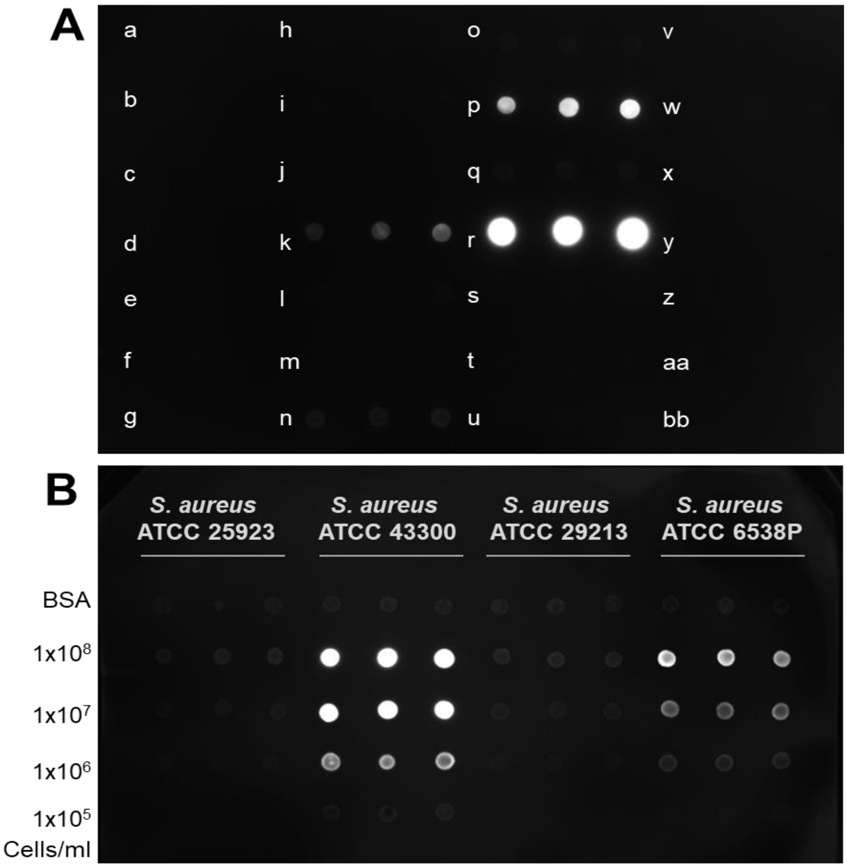

Figure S2. MBL binding test with various bacteria. (A) Image confirming the combination of purified MBL and various bacteria including (a) D.W, (b) LB, (c) BSA, (d) *E.coli* (KCTC 1116), (e) *E.coli* (ATCC 25922), (f) *S. flexneri* (ATCC 29903), (g) *A. baumannii* (ATCC 19606), (h) *K. pneumoniae* (ATCC 13883), (i) *K. pneumoniae* (ATCC 700603), (j) *P. aeruginosa* (ATCC 10145), (k) *P. aeruginosa* (ATCC 27853), (l) *B. cereus* (ATCC 14579), (m) *S. enterica* (ATCC 15277), (n) *V. vulnificus* (ATCC 27562), (o) *S. aureus* (ATCC 25923), (p) MSSA (*S. aureus*, ATCC6538P), (q) *S. aureus* (ATCC 29213), (r) MRSA (*S. aureus* ATCC 43300), (s) *S. haemolyticus* (ATCC 29970), (t) *S. saprophyticus* (ATCC 15305), (u) *S. epidermidis* (ATCC14990), (v) *E. faecalis* (ATCC 19433), (w) *E. faecalis* (ATCC 29212), (x) *E. faecalis* (ATCC 51299), (y) *E. faecium* (ATCC 19434), (z) *E. faecium* (ATCC 700221), (aa) *S. pneumonia* (ATCC 33400), (bb) *B. parapertussis* (ATCC 15311). (B) Image confirming the degree of binding and detection limit of MBL for 4 types of *S. aureus* using

dot blot

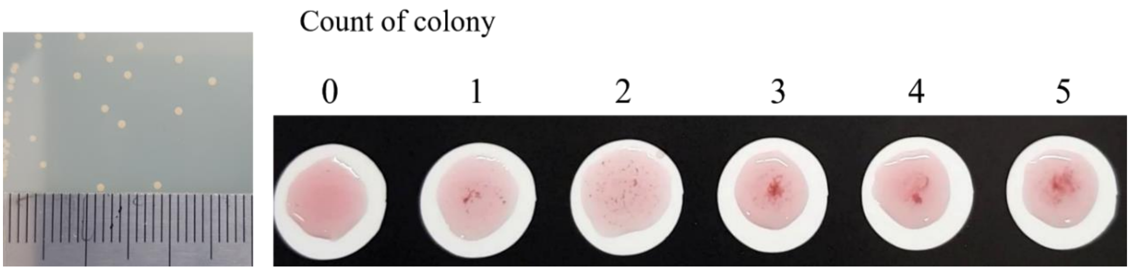

Figure S3. Image confirming the degree of aggregation of MRSA probe according to the number of colonies having a diameter of about 1 mm.

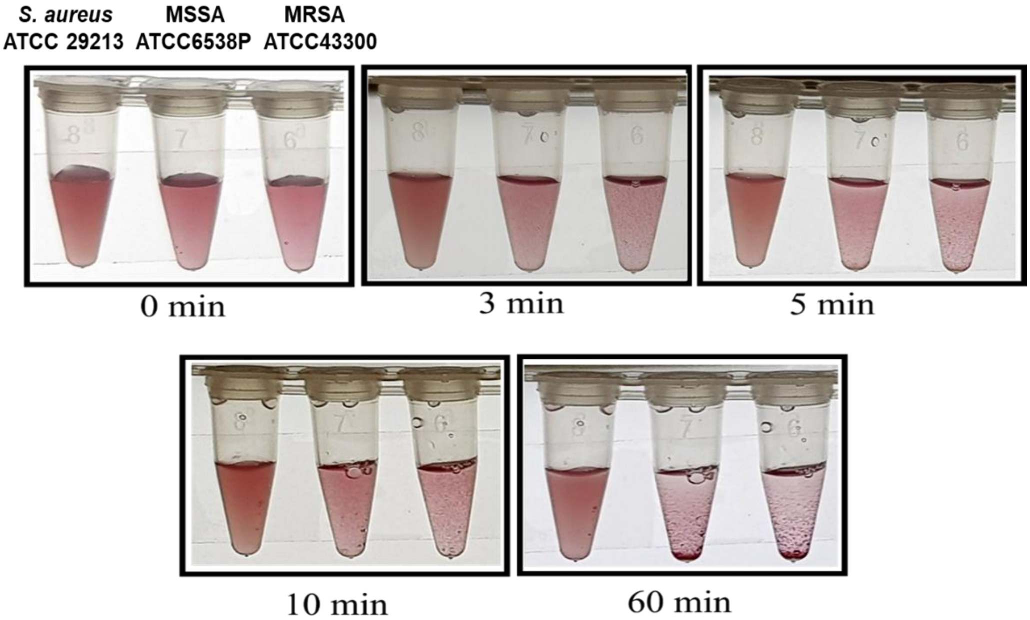

Figure S4. Images showing the degree of sedimentation over time using the MRSA probe.

Supplemental References

(1) Yang, X.; Dang, Y.; Lou, J.; Shao, H.; Jiang, X. *Theranostics* **2018**, *8*, 1449-1457.

(2) Tiet, P.; Clark, K. C.; McNamara, J. O., 2nd; Berlin, J. M. *Bioconjug Chem.* **2017**, *28*, 183-193.
